# Assessment of a serum calcification propensity test for the prediction of all-cause mortality among hemodialysis patients

**DOI:** 10.1186/s12882-023-03069-6

**Published:** 2023-02-15

**Authors:** Adam M Zawada, Melanie Wolf, Abraham Rincon Bello, Rosa Ramos-Sanchez, Sara Hurtado Munoz, Laura Ribera Tello, Josep Mora-Macia, M. Amparo Fernández-Robres, Jordi Soler-Garcia, Josep Aguilera Jover, Francesc Moreso, Stefano Stuard, Manuela Stauss-Grabo, Anke Winter, Bernard Canaud

**Affiliations:** 1 Fresenius Medical Care Deutschland GmbH, Else-Kroener-Str. 1, 61352 Bad Homburg, Germany; 2 Fresenius Medical Care España, S.A, Tres Cantos, Madrid, Spain; 3 Fresenius Medical Care Services Cataluña, S.L, Barcelona, Spain; 4 School of Medicine, University of Montpellier, Montpellier, France; 5 Fresenius Medical Care Services Cataluña, S.L, Tarragona, Spain

**Keywords:** Calcification, Hemodialysis, Hospitalization, Mortality risk, T50

## Abstract

**Background:**

Vascular calcification is a major contributor to the high cardiac burden among hemodialysis patients. A novel in vitro T50-test, which determines calcification propensity of human serum, may identify patients at high risk for cardiovascular (CV) disease and mortality. We evaluated whether T50 predicts mortality and hospitalizations among an unselected cohort of hemodialysis patients.

**Methods:**

This prospective clinical study included 776 incident and prevalent hemodialysis patients from 8 dialysis centers in Spain. T50 and fetuin-A were determined at Calciscon AG, all other clinical data were retrieved from the European Clinical Database. After their baseline T50 measurement, patients were followed for two years for the occurrence of all-cause mortality, CV-related mortality, all-cause and CV-related hospitalizations. Outcome assessment was performed with proportional subdistribution hazards regression modelling.

**Results:**

Patients who died during follow-up had a significantly lower T50 at baseline as compared to those who survived (269.6 vs. 287.7 min, p = 0.001). A cross-validated model (mean c statistic: 0.5767) identified T50 as a linear predictor of all-cause-mortality (subdistribution hazard ratio (per min): 0.9957, 95% CI [0.9933;0.9981]). T50 remained significant after inclusion of known predictors. There was no evidence for prediction of CV-related outcomes, but for all-cause hospitalizations (mean c statistic: 0.5284).

**Conclusion:**

T50 was identified as an independent predictor of all-cause mortality among an unselected cohort of hemodialysis patients. However, the additional predictive value of T50 added to known mortality predictors was limited. Future studies are needed to assess the predictive value of T50 for CV-related events in unselected hemodialysis patients.

**Supplementary Information:**

The online version contains supplementary material available at 10.1186/s12882-023-03069-6.

## Background

Patients with stage 5 chronic kidney disease on dialysis (CKD5HD) have an exceedingly high risk of morbidity and mortality [1]. Vascular calcification as cause for vascular stiffness is a major contributor to the high cardiac burden among this patient population [2].

Calcium and phosphate accumulation in the tunica media of arteries and active vascular cell function modifications induced by such deposits are the underlying mechanisms of vascular calcification [3]. This biomineralization, namely vascular ossification process [4] is strongly regulated by promoters and inhibitors of calcification. In human blood, the formation of so called primary calciprotein particles (CPPs) prevents the precipitation of supersaturated calcium and phosphate [5–8]. These CPPs mainly consist of non-crystalline calcium and phosphate, bound to fetuin-A and other proteins, and are part of the mineral buffering system inherent in blood [8]. Spontaneous rearrangements within these primary CPPs lead to formation of secondary CPPs, which are larger and contain crystalline hydroxyapatite [7, 8]. The half-maximal transformation time from primary to secondary CPPs is called T50 and reflects the capacity of serum to resist crystallization of calcium and phosphate [9]. Importantly, T50 is dependent on various other calcification-related factors such as magnesium, phosphate, calcium, bicarbonate, fetuin-A or albumin [9].

A recently developed test (T50-test) can determine this T50 time and thus reflects the patients’ capacity to resist calcification [10]. A longer T50 time indicates a higher calcification resistance of the patients’ serum. Previous clinical and epidemiological studies identified baseline T50 as predictor of all-cause and cardiovascular (CV)-related mortality among CKD patients, renal transplant patients and among a selected cohort of hemodialysis patients [11–15]. However, the PREVEND study (Prevention of Renal and Vascular End Stage Disease) has recently found that Serum T50 was associated with cardiovascular mortality in general population but not with all-cause mortality after adjustment [16].

In the present study, we now evaluated whether these results can be confirmed in an unselected cohort of CKD5HD patients and whether T50 may predict future hospitalizations among these patients.

## Methods

### Study population

In September / October 2017 we screened hemodialysis patients in 8 NephroCare centers in Catalonia, Spain (Centro de Diálisis Barcelona – Rosselló, Centro de Diálisis Barcelona – Diagonal, Centro de Diálisis Barcelona – Glories, Centro de Diálisis Granollers, Centro de Diálisis Hospitalet, Centro de Diálisis Reus, Centro de Diálisis Tarragona, Centro de Diálisis Terrassa) for inclusion in the present prospective clinical study. Adult incident and prevalent hemodialysis patients, being treated three times per week were eligible for study inclusion. Exclusion criteria were predefined as any condition which could interfere with the patients’ ability to comply with the study: pregnancy and participation in other calcification related clinical studies during the preceding 30 days. All patients gave written informed consent to participate in the clinical study and the study was approved by the ethics committee (Comité Ético de Investigación Clínica del Hospital Clínic de Barcelona, code: HD-T50-01-ES). The study was performed in adherence with the Declaration of Helsinki. The study is registered at clinicaltrials.gov (NCT03292029, registration on 25/09/2017).

In 782 patients who agreed to participate, one additional blood sample was taken prior to their dialysis treatment during the routine semestral blood sampling between October 2nd and October 5th, for the determination of T50. 5 patients were excluded for the primary analysis as they did not meet inclusion criteria (not treated 3 times per week) and one additional patient due to labeling problems of the blood sample, yielding 776 patients for the primary analysis.

### Data source

T50 and fetuin-A were determined at Calciscon AG (Nidau, Switzerland) according to Pasch et al. [10]. All other clinical and laboratory information were retrieved from the European Clinical Database (EuCliD) and linked to T50 and fetuin-A data using an anonymous study code. EuCliD is a clinical information system, implemented across all NephroCare clinics; routinely collected medical information is available in EuCliD as described before [17, 18]. These data include demographic information, comorbidities, laboratory data, medication as well as information on underlying kidney disease, vascular access, dialysis treatments and clinical outcome (hospitalization and mortality). ICD-10 (International Classification of Diseases, version 10) codes are used to classify disease information.

### Exposure definition

Baseline T50 measurement was defined as exposure. The index date of the study (baseline) was defined as the time point of blood sampling for T50 and fetuin-A determination.

In additional analyses, levels of a set of six calcification related parameters (albumin, phosphate, calcium, magnesium, bicarbonate, and fetuin-A) were defined as exposure. For the six calcification related parameters no imputation was performed if missing at index date.

### Outcome assessment

Time to all-cause mortality was predefined as primary outcome. Time to cardiovascular (CV)-related mortality, time to all-cause hospitalization and time to CV-related hospitalization as secondary outcomes. All mortality and hospitalization information including the date of death/hospitalization during follow-up and cause of death/hospitalization was retrieved from EuCliD. CV-related mortality was defined as any death reason with ICD-10 code I00-I99.

All patients were followed from the index date until death, kidney transplantation, center change, treatment stop, spontaneous recovery, loss to follow-up or end of study (two years after baseline blood sampling), whichever arose first.

### Baseline patient and treatment characteristics

Baseline demographic patient information was calculated as of index date and information from the preceding 3 months was used if not available at index date. Baseline information included demographic information, comorbidities, vascular access, body composition, blood pressure, medications, treatment (modality, treatment time, OCM Kt/V, dialysate electrolyte prescription, convective volume) and laboratory (T50, fetuin-A, albumin, phosphate, calcium, magnesium, bicarbonate, creatinine, hemoglobin, C-reactive protein, alkaline phosphatase, PTH) data.

Dialysis vintage was calculated from the date of first dialysis until index date. Body mass index (BMI) was calculated by using the post-dialytic weight. The coding algorithm proposed by Quan et al. was used to classify comorbidities and calculate the Charlson Comorbidity Index (CCI) as well as the age-adjusted CCI [19, 20]. Accordingly, baseline cardiovascular disease was defined as myocardial infarction, congestive heart failure, peripheral vascular disease and cerebrovascular disease. Hydration status was assessed with the Body Composition Monitor (BCM). Patients with hydration status ≤-1.1 L were classified as fluid depleted, patients with hydration status > 1.1 L as fluid overload and patients with hydration status between − 1.1 and 1.1 L as normohydrated, as suggested before [21, 22]. Pulse pressure was calculated as the difference between systolic and diastolic blood pressure values averaged across pre- and post-dialytic values from three consecutive treatment days with complete blood pressure data starting at baseline. Albumin was measured with bromocresol green [23]. Medication information was presented for statins, phosphate binders and agents acting on the renin-angiotensin system.

### Statistical analyses

Descriptive statistics were calculated for patient and treatment characteristics as of index date. The performance of baseline T50 values in predicting the primary endpoint was examined using time-to-event methodology in combination with predictive analytics. We applied proportional subdistribution hazards regression modelling in the presence of competing risks [24] treating kidney transplantation before the endpoint of interest as competing events in every model. All other drop-out reasons were censored. For the secondary CV-related outcomes all other reasons for death or hospitalization were also treated as competing events for the corresponding outcome. A two-degree fractional polynomial (FP2) was assumed to allow for a potentially nonlinear relationship between T50 and the subdistribution hazard of all-cause death [25]. Model selection was based on the “RA2” function selection algorithm with level α chosen as 0.15 [26]. Each comparison was based on the difference in model deviances and the corresponding p-value resulted from a likelihood ratio test based on an approximate Chi-Square distribution. Stratified k-fold cross-validation was applied for internal model validation [27] ensuring equal distributions of event statuses (event of interest, competing event, censoring) across the k datasets. Here, an arbitrary run j∈\{1,…,k} used the respective “left-out fold” dataset j as validation and the data combining all remaining sets as derivation data. For the primary analysis, we chose k = 10 following the recommendations in the literature [28].

The predictive performance of each of the k models was analyzed in terms of discrimination and calibration. To evaluate calibration the cumulative incidence estimates in the validation dataset were computed by the non-parametric Aalen-Johansen estimator [29]. Discrimination was evaluated by an adapted version of Harrell’s c-index accounting for competing risks [30] in the derivation dataset.

In a sensitivity analysis, we repeated the aforementioned procedures to assess the additional predictive value of T50 after the inclusion in the model of other known mortality predictors such as age, gender, CCI, vascular access, dialysis vintage and modality.

In a similar manner, we also performed additional analyses regarding the secondary time-to-event outcomes as well as using the six calcification related parameters as the exploratory variables. Due to the limited number of CV events, cross-validation was performed using k = 5 for all additional analyses.

All statistical analyses were performed using the SAS® statistical software (Version 9.4). An adapted version of the publicly available and documented %mfp8 SAS macro [31] was developed for the RA2 function selection algorithm in the context of Fine and Gray incorporating FP2.

## Results

776 patients were included in the present analysis. Demographic characteristics as of index date are presented in Table 1. Mean age of the patients was 72 years and 63.8% were men. Diabetes mellitus (19.9%), glomerular diseases (11.0%) and hypertension/atherosclerosis (9.8%) were the most frequently documented causes of end-stage renal disease. Patients had a mean dialysis vintage of 35.1 months, 42.5% had diabetes and 55.0% cardiovascular disease. Mean overhydration was 1.6 L and 60.3%, 34.5% and 1.7% of patients were overhydrated, normohydrated and underhydrated, respectively (3.5% missing information on hydration status). Most patients were treated with hemodiafiltration (93.6%) and fistula was the most common vascular access (69.6%), 52.7% of the patients were on statins and 69.6% on phosphate binders (56.7% calcium-based, 27.5% non calcium-based phosphate binders, calculated among the total population).

**Table 1:** Baseline characteristics of the total study population

Parameter^1^	Total population (n = 776)	T50 91-255 min (n = 259)	T50 256-309 min (n = 261)	T50 310-544 min (n = 256)	p-value^1^
Age [years]	72 ± 13	73 ± 11	73 ± 13	70 ± 14	0.0082
Male [%]	63.8	66.0	60.9	64.5	0.4633
Dialysis vintage [months]	35.1 [15.4;71.2]	35.4 [17.2;68.0]	34.2 [14.5;76.8]	35.9 [14.7;71.2]	0.9993
Causes of end-stage renal disease [%]					0.6201
Diabetes mellitus	19.9	21.2	21.1	17.2	
Glomerular disease	11.0	11.2	8.8	12.9	
Hypertension/atherosclerosis	9.8	12.7	7.7	9.0	
Congenital malformations	5.4	4.6	6.9	4.7	
Interstitial kidney diseases	5.0	4.6	5.4	5.1	
Cancer	0.9	0.4	1.2	1.2	
Amyloidosis	0.4	0.4	0.8	0.0	
Other	1.8	1.9	1.2	2.3	
Unknown	45.9	42.9	47.1	47.7	
Height [cm]	162.6 ± 9.6	163.0 ± 9.9	161.9 ± 9.5	162.9 ± 9.4	0.3191
Body weight, postdialysis [kg]	70.9 ± 15.7	71.4 ± 15.4	70.1 ± 15.6	71.2 ± 16.2	0.6047
Diabetes [%]	42.5	45.2	39.1	43.4	0.3529
Cardiovascular disease [%]	55.0	59.5	50.6	55.1	0.1257
Age adjusted Charlson comorbidity index	7.2 ± 2.3	7.3 ± 2.3	7.2 ± 2.4	6.9 ± 2.4	0.0595
*BCM measurements*					
Body mass index [kg/m²]	26.8 ± 5.5	26.9 ± 5.2	26.7 ± 5.5	26.8 ± 5.7	0.9641
Total body water [l]	32.9 ± 7.0	33.4 ± 7.6	32.6 ± 6.8	32.9 ± 6.7	0.4479
Intracellular water [l]	15.9 [13.5;18.8]	15.9 [13.4;18.9]	15.7 [13.3;18.4]	16.0 [13.7;18.9]	0.6516
Extracellular water [l]	16.5 ± 3.1	16.7 ± 3.0	16.4 ± 3.2	16.4 ± 3.2	0.4811
Fat tissue mass [kg]	28.3 ± 11.1	28.4 ± 10.8	27.7 ± 10.9	28.6 ± 11.7	0.6505
Lean tissue mass [kg]	30.1 [24.1;37.1]	30.7 [23.7;37.2]	30.1 [23.8;37.0]	29.9 [24.6;37.0]	0.8358
Fluid overload [l]	1.6 ± 1.3	1.6 ± 1.3	1.6 ± 1.3	1.4 ± 1.3	0.2252
Systolic blood pressure, predialysis [mmHg]	137.3 ± 27.6	136.2 ± 29.0	136.4 ± 25.5	139.2 ± 28.1	0.3684
Diastolic blood pressure, predialysis [mmHg]	63.3 ± 16.1	62.8 ± 16.5	62.7 ± 15.8	64.4 ± 16.0	0.4261
Pulse pressure [mmHg]	75.2 ± 20.8	75.4 ± 22.8	75.0 ± 19.1	75.2 ± 20.3	0.9791
*Vascular access [%]*					
Fistula	69.6	67.2	70.5	71.1	0.6508
Graft	3.1	3.1	2.3	3.9	
Catheter	27.3	29.7	27.2	25.0	
*Treatment modality [%]*					
Hemodialysis	6.4	7.0	3.8	8.6	0.0810
Hemodiafiltration (HDF)	93.6	93.1	96.2	91.4	
*Treatment parameters*					
Effective treatment time [min]	242.0 ± 11.9	241.5 ± 11.7	242.2 ± 7.9	242.4 ± 15.0	0.6744
OCM Kt/V^2^	1.9 ± 0.4	1.9 ± 0.4	2.0 ± 0.4	1.9 ± 0.4	0.1968
Convective volume (HDF) [l]	26.3 [24.2;28.1]	26.0 [24.0;27.7]	26.2 [24.5;28.1]	26.5 [24.8;28.6]	0.0335
*Dialysate electrolyte prescription*					
Bicarbonate (acetic acid) [mmol/l]	35.0 [35.0;35.0]	35.0 [35.0;35.0]	35.0 [35.0;35.0]	35.0 [35.0;35.0]	1.0000
Calcium [mmol/l]	1.5 [1.4;1.5]	1.5 [1.5;1.5]	1.5 [1.3;1.5]	1.5 [1.5;1.5]	0.0384
Potassium [mmol/l]	1.5 [1.5;2.0]	1.5 [1.5;2.0]	1.5 [1.5;2.0]	1.5 [1.5;1.5]	0.0117
Sodium [mmol/l]	140.0 [140.0;140.0]	140.0 [140.0;140.0]	140.0 [140.0;140.0]	140.0 [140.0;140.0]	1.0000
Magnesium [mmol/l]	0.5 [0.5;0.5]	0.5 [0.5;0.5]	0.5 [0.5;0.5]	0.5 [0.5;0.5]	1.0000
*Lab values*					
T50 CIT^2^ [min]	283.4 ± 64.0	215.9 ± 32.5	282.8 ± 16.0	352.2 ± 41.6	< 0.0001
Fetuin-A [mg/ml]	0.4 ± 0.1	0.3 ± 0.1	0.4 ± 0.1	0.4 ± 0.1	< 0.0001
Albumin [g/dl]	3.7 ± 0.4	3.6 ± 0.4	3.7 ± 0.3	3.8 ± 0.3	< 0.0001
Phosphate [mmol/l]	1.4 ± 0.4	1.6 ± 0.4	1.4 ± 0.4	1.3 ± 0.3	< 0.0001
Calcium [mmol/l]	2.2 ± 0.1	2.2 ± 0.1	2.2 ± 0.1	2.3 ± 0.1	< 0.0001
Magnesium [mmol/l]	0.9 ± 0.2	0.9 ± 0.1	0.9 ± 0.1	1.0 ± 0.2	< 0.0001
Bicarbonate [mmol/l]	23.5 ± 2.7	22.5 ± 2.6	23.2 ± 2.3	24.8 ± 2.6	< 0.0001
Creatinine, predialysis [mg/dl]	6.9 ± 2.0	7.1 ± 2.0	6.8 ± 2.0	6.9 ± 1.9	0.1716
Hemoglobin [g/dl]	10.9 ± 1.2	11.0 ± 1.3	10.9 ± 1.2	10.9 ± 1.2	0.7025
C-reactive protein [mg/l]	6.6 [3.2;13.7]	8.6 [4.1;22.2]	5.8 [3.0;12.6]	5.9 [2.8;10.7]	< 0.0001
iPTH [pg/ml]	225.2 [136.1;361.6]	237.7 [128.1;399.4]	230.1 [135.7;349.1]	216.7 [144.2;333.6]	0.4695
Alkaline phosphatase [IU/l]	92.0 [70.0;121.0]	91.0 [69.0;122.0]	92.0 [70.5;120.0]	92.0 [74.0;119.0]	0.8629
*Medications* ^*3*^ *[%]*					
Statins	52.7	52.9	52.9	52.3	0.9900
Phosphate binders	69.6	73.0	68.2	67.6	0.3449
Calcium-based phosphate binders^4^	56.7	58.3	54.4	57.4	0.6427
Non-calcium-based phosphate binders^4^	27.5	30.1	28.0	24.2	0.3163
Agents acting on the renin-angiotensin system	19.9	19.7	19.9	19.9	0.9971
Vitamin D	46.4	44.8	44.4	50.0	0.3668
Calcifediol (25-OH-D3)^4^	2.1	1.5	3.1	1.6	0.3752
Calcimetics	19.1	22.0	16.9	18.4	0.3074

^*1*^ *Data are presented as mean ± SD, median [q1;q3] or n [%], as appropriate. P-value was calculated by one-way ANOVA/Kruskal-Wallis for continuous variables and chi-squared test for categorical variables*

^*2*^ *OCM: Online Clearance Monitoring; CIT: Calcification Inhibition Test*

^*3*^ *ATC codes for medications: statins (C10AA, C10BA, C10BX), phosphate binders (V03AE17, V03AE07, A12AA12, V03AE16, A12AA04, V03AE03, V03AE04, V03AE02, V03AE05, V03AE06, V03AE08, V03AE11, V03AE12), agents acting on the renin-angiotensin system (C09AA, C09BA, C09BB, C09BX, C09CA, C09DA, C09DB, C09DX, C09XA), Vitamin D (A11CC, A12AX), Calcimetics (H05BX01).*

^*4*^ *Percentage was calculated among the total population*

Mean T50 was 283.4 min; no statistical differences in T50 were found in subgroups of patients, when stratifying according to dialysis modality (hemodialysis: 293.3 ± 69.3; hemodiafiltration: 282.7 ± 63.6, p = 0.2963), vascular access (fistula/graft: 284.2 ± 61.4; catheter: 281.2 ± 70.6; p = 0.5851) and hydration status (normo-/underhydrated: 286.3 ± 63.3; overhydrated: 282.7 ± 64.2; p = 0.4606).

When stratifying patients in tertiles according to their T50 values, patients with highest T50 values were younger (p = 0.0082), had higher convective volume (p = 0.0335) and had a different profile in calcification related lab values (Table 1). Most other parameter such as gender, dialysis vintage, comorbidities, body composition, vascular access or medications did not differ significantly between the three groups.

During follow-up, 185 (23.8%) patients died, 63 (8.1%) due to cardiovascular complications. Further causes of death were: sepsis (22 [2.8%]), cancer (15 [1.9%]), diseases of the respiratory system (13 [1.7%]) and diseases of the digestive system (7 [0.9%]). For 65 (8.4%) patients the death reason was not specified. 112 (14.4%) patients were transplanted, 17 (2.2%) changed the dialysis center, 9 (1.2%) stopped the treatment and 2 (0.3%) were lost to follow-up. Thus, 451 (58.1%) patients were followed until the end of the two-year follow-up. During follow-up, 483 (62.2%) patients were at least once hospitalized, 88 (11.3%) due to cardiovascular reasons.

Mean T50 was significantly lower in patients who died during follow-up as compared to patients who survived (mean T50: 269.6 vs. 287.7 min, p = 0.001). In line, in cumulative incidence analyses, patients in lowest T50 tertile had the highest probability to die, followed by the middle and the highest T50 tertile (Figure S1). Moreover, 10-fold cross-validation consistently identified T50 as significant linear predictor of all-cause mortality with crude subdistribution hazard ratios (sHR) varying between 0.9954 and 0.9961 (per min) and an averaged c-statistic of 0.5767 (Table 2).

**Table 2:** T50 and all-cause mortality

Run	Model	Crude sHR^1^	95% CI_sHR_^2^	c statistic
1	Linear	0.9955	[0.9931; 0.9980]	0.5779
2	Linear	0.9954	[0.9928; 0.9980]	0.5815
3	Linear	0.9957	[0.9932; 0.9983]	0.5758
4	Linear	0.9958	[0.9931; 0.9984]	0.5717
5	Linear	0.9957	[0.9932; 0.9982]	0.5745
6	Linear	0.9956	[0.9930; 0.9981]	0.5828
7	Linear	0.9958	[0.9932; 0.9984]	0.5775
8	Linear	0.9955	[0.9930; 0.9980]	0.5818
9	Linear	0.9961	[0.9935; 0.9988]	0.5688
10	Linear	0.9958	[0.9933; 0.9984]	0.5742

^1^sHR = subdistribution hazard ratio, estimated from a Fine & Grey regression including T50 variables as independent variable

^2^CI = confidence interval of subdistribution hazard ratio

In sensitivity analysis, we investigated the additional predictive value of T50 after the inclusion in the model of known mortality predictors in this context. The multivariable model without T50 yielded an averaged c-statistic for all-cause mortality of 0.685. The respective sHRs are provided in **Table S1**. Addition of T50 into this model further improved c-statistic (0.6919); notably, across all cross-validation runs, T50 was consistently selected as a linear predictor (Table 3).

**Table 3:** Known mortality predictors & T50 and all-cause mortality

Variable	Number of runs	Times variable selected	Number of linear fits	Adjusted sHR [CI_sHR_]^1^ range^2^	
				min	max
Age [years]	10	10	10	1.0429 [1.0269; 1.0592]	1.0542 [1.0371; 1.0717]
CCI^3^	10	10	10	1.0960 [1.0069; 1.1930]	1.1688 [1.0742; 1.2718]
Gender [ref male]	10	10	10	1.4290 [1.0167; 2.0086]	1.6036 [1.1421; 2.2516]
Vascular access [ref catheter]	10	10	10	0.6014 [0.4305; 0.8402]	0.6844 [0.4835; 0.9688]
Dialysis vintage [month]	10	10	4	1.0036 [1.0018; 1.0053]	1.0046 [1.0029; 1.0063]
Dialysis modality [ref HD]	10	2	2	1.6404 [0.8388; 3.2078]	1.6997 [0.8029; 3.5983]
T50 [min]	10	10	10	0.9961 [0.9937; 0.9990]	0.9971 [0.9944; 0.9999]

^1^CI_sHR_ = confidence interval of subdistribution hazard ratio

^2^only indicated for linear models

^3^CCI = Charlson comorbidity index

Regarding secondary outcomes, 5-fold cross-validation did not identify T50 as a predictor for CV-related mortality and CV-related hospitalizations (Table S2). However, 10-fold cross-validation identified T50 as significant linear predictor of all-cause hospitalization with subdistribution hazard ratios varying between 0.9976 and 0.9982 throughout 7 out of 10 validation runs (mean c statistic: 0.5284).

Finally, we explored the association of the set of six calcification related parameters (albumin, phosphate, calcium, magnesium, bicarbonate, fetuin-A) with all-cause mortality and all-cause hospitalization (Table S3), and whether T50 may improve the ability to predict outcomes (Table S4). Due to the low number of CV-related events and high number of variables within the model, no analyses regarding CV-related endpoints were performed. The cross-validated analysis yielded an averaged c-statistic for all-cause mortality of 0.6211; here, albumin was consistently selected as significant linear predictor in the subdistribution hazards model with sHRs between 0.2652 and 0.3736. Only within one run, higher calcium (sHR: 3.77) and lower fetuin-A (sHR: 0.013) levels were additionally linearly associated with an increased incidence of all-cause mortality. Phosphate, magnesium and bicarbonate were not selected as significant linear predictors (Table S3). Finally, addition of T50 to the model provided no additional prognostic value (Table S4). Comparable results were found for all-cause hospitalization, with however lower discrimination.

## Discussion

In the present clinical study, we identified T50 as an independent predictor of all-cause mortality among hemodialysis patients. The linear association was consistent across all ten cross validation runs and remained significant in sensitivity analyses when including known mortality predictors. Prediction accuracy was however in need for improvement. This was also the case for the prediction of all-cause hospitalization, where T50 was identified as linear predictor in most cross validation runs. Regarding CV-related events, T50 did not predict mortality and hospitalizations in our models; the low number of CV-related events could have precluded identification of potential associations.

Traditional cardiovascular risk factors cannot fully explain the exceedingly high morbidity and mortality rate of dialysis patients [32]. Further non-traditional risk factors are highly prevalent in dialysis patients and significantly add to the high burden of these patients. Here, chronic kidney disease–mineral bone disorder (CKD-MBD) is a central component in the pathogenesis of cardiovascular disease among dialysis patients [33]. Disturbances in mineral and bone metabolism lead to accelerated vascular calcification, a process which is triggered by the osteogenic transdifferentiation of vascular smooth muscle cells towards a pro-calcifying state leading to arterial stiffness and hemodynamic changes [34]. As shown in various studies, increase in arterial stiffness due to vascular calcification may precipitate cardiovascular complications such as increased cardiac afterload, left ventricular hypertrophy and heart failure [35–38]. In line, the extend of calcifications has been associated with mortality rate in dialysis patients [39].

The precipitation of calcium and phosphate in human blood is a central step in the vascular calcification process. In blood, calcium and phosphate forms together with liver-derived plasma protein fetuin-A and other proteins primary calciprotein particles (CPPs), which prevent the precipitation of calcium and phosphate and are part of the mineral buffering system of blood [8]. The formation of secondary CPPs, composed of crystalline calcium and phosphate, depend on the interplay of calcification promoters and inhibitors which are available in the human blood. Importantly, CPPs may contribute to chronic inflammation, premature ageing phenotype and vascular remodeling [40]. Here, experimental studies demonstrated that especially those secondary CPPs have the potential to induce inflammation and calcification in cultured cells, while primary CPPs are less potent in cellular activation [8, 41, 42]. Thus, a longer transition time from primary to secondary CPPs in human blood is linked with less cell activation and osteogenic transdifferentiation in the body. Due to the association of secondary CPPs with mortality risk factors, such as inflammation and/or cellular activation, a longer transition time from primary to secondary CPPs could also have a beneficial effect for patients beyond the classical pathway of vascular calcification.

The recently developed T50-test can determine the transition time T50 and therefore the calcification inhibition potential of human blood [10]. For this test, patients’ serum is challenged with supersaturated calcium and phosphate solutions which leads to formation of primary CPPs in vitro. This solution is incubated at 37 °C and the spontaneous formation of secondary CPPs is measured with nephelometry. As the T50 value is determined by many calcification promoting and inhibiting factors present in patients’ blood, the results of the test provide an integrated view on the calcification inhibitory system.

The value of T50 in terms of mortality prediction was investigated in several studies before. One study comprised 184 patients with CKD stages 3 and 4 and identified T50 as predictor of all-cause mortality, even after adjustment for demographic, biochemical, renal and cardiovascular covariates (HR: 2.24 [1.13; 5.37], for lowest vs. highest T50 tertile) [15]. A recent analysis with 3404 CKD patients (eGFR 20–70 ml/min/1.73 m^2^) from the Chronic Renal Insufficiency Cohort Study (CRIC) partly confirmed the previous results in CKD patients: here, lower T50 was also significantly associated with higher risk for all-cause mortality (HR: 1.16 [1.09; 1.24], per SD decrease). However, after adjustment for eGFR and 24-hour urinary protein, the association was lost [14]. Two studies with renal transplant patients (n = 699 and n = 1435) also investigated the association between T50 and cardiovascular-related mortality [12, 13]: both studies found T50 to be independently associated with all-cause (HR: 1.43 [1.11; 1.85], per SD decrease and 1.60 [1.00; 2.57], for lowest vs. highest T50 quartile, respectively) as well as cardiovascular-related mortality (HR: 1.55 [1.04 to 2.29], per SD decrease and 3.60 [1.10; 11.83], for lowest vs. highest T50 quartile, respectively). In hemodialysis patients, Pasch et al. [11] performed a post hoc analysis of the EVOLVE (Evaluation of Cinacalcet Therapy to Lower Cardiovascular Events) trial and measured T50 in 2785 baseline samples. In these patients with secondary hyperparathyroidism the authors found lower T50 to be independently associated with a higher risk for all-cause mortality (HR: 1.10 [1.02; 1.17], per SD decrease) and the composite endpoint, consisting of all-cause mortality, myocardial infarction, hospitalization for unstable angina, heart failure and peripheral vascular event (HR: 1.15 [1.08; 1.22], per SD decrease). Another recent study among incident hemodialysis patients (n = 388) failed to find an association between baseline T50 and risk for mortality [43].

Our present study confirmed previous findings in an unselected hemodialysis cohort regarding the primary endpoint all-cause mortality. 10-fold cross-validation consistently characterized T50 as a linear predictor of all-cause mortality. However, the additional predictive value of T50 added to known mortality predictors in these patients was limited. Our models could not confirm T50 as predictor of cardiovascular-related mortality. This appears surprisingly given the stronger association of T50 with cardiovascular-related mortality then with all-cause mortality in the previous studies. However, it is important to note that our study was powered for the primary (all-cause mortality) but not the secondary endpoints. Thus, the low number of cardiovascular-related events could have precluded identification of potential associations here. Further studies are needed to confirm previous findings in unselected cohorts of dialysis patients.

Against the background that most large interventional trials failed to improve survival in hemodialysis patients, novel approaches are clearly needed to reduce the high morbidity and mortality burden in these patients. Importantly, T50 has previously been shown to be therapeutically modifiable in patients with kidney disease [8, 9, 44]. However, to date no interventional studies are available demonstrating a positive clinical effect following a T50-improving therapy. To close this gap, prospective clinical trials are ongoing which shall test the effects of dialysis, citrate, bicarbonate, phosphate lowering, magnesium and vitamin K on T50 [8]. Results of these studies will help to better understand the pathogenesis of calcification and may open new doors for therapeutical interventions in dialysis patients.

Our study has several strengths. It is the first study which evaluated the predictive value of single T50 measurements in a large cohort of unselected hemodialysis patients. This study was implemented in routine care setting and thus provides a view into real-world setting, while exploring clinically relevant outcomes. One major strength of our study was the application of complex and powerful statistical methodology, which goes beyond existing context-related approaches. To the best of our knowledge, our study was the first approach to combine fractional polynomials and Fine and Gray regression modeling.

Several limitations of our study shall be considered when interpreting our findings. First, the study was powered for the primary endpoint all-cause mortality, which resulted in good estimation and model stability regarding the primary endpoint and sensitivity analyses. Thus, the results obtained from the secondary analyses should be treated with caution and further investigations are necessary to draw definite conclusions. We also acknowledge that the c-statistic alone to assess model improvement has its limitations. However, this is a very common and well-established tool to predict an outcome [45]. Cause of death could not be verified with death certificates in the present study, and therefore the percentage of CV-related mortality could be underestimated. Moreover, we performed single T50 measurements at baseline and did not analyze longitudinal changes of T50. Previous studies indicate that changes in T50 levels over time may have a better prognostic value as compared to single T50 measurements [46]. Finally, T50 may not be a single contributor and various calcification-related factors such as magnesium, phosphate, calcium, bicarbonate, fetuin-A or albumin may play a role in vascular calcification processes. However, in our analysis, only albumin was consistently selected as significant linear predictor for all-cause mortality among these calcification-related parameters.

## Conclusion

In conclusion, the present study identified T50 as an independent predictor of all-cause mortality among an unselected cohort of hemodialysis patients. However, the additional predictive value of T50 added to known mortality predictors in these patients was limited. Future studies are needed to assess the predictive value of T50 for CV-related events in unselected hemodialysis patients.

## Electronic supplementary material

 Below is the link to the electronic supplementary material.

Supplementary Material 1

## Data Availability

The EuCliD database is a comprehensive electronic therapy information system which has been established within the framework of Fresenius Medical Care’s quality improvement and management programs in NephroCare clinics. Data from this database cannot be shared publicly. Please contact the corresponding author for more information.
