# Supplementary material for: Assessment of a serum calcification propensity test for the prediction of all-cause mortality among hemodialysis patients
Source: BMC Nephrol. 2023 Feb 15;24:35. doi: 10.1186/s12882-023-03069-6 (PMC9933331; doi:10.1186/s12882-023-03069-6)
Supplement: Supplementary file 1 — Supplementary Material 1 [file 12882_2023_3069_MOESM1_ESM.docx]

**Table of Content**

Supplemental Table S1: Known mortality predictors and all-cause mortality

Supplemental Table S2: T50 and secondary outcomes

Supplemental Table S3: Calcification related parameters and outcomes

Supplemental Table S4: Calcification related parameters & T50 and outcomes

Supplemental Figure S1: Cumulative Incidence Function for T50 groups

**Table S1. Known mortality predictors and all-cause mortality**

| **Variable** | **Number of runs** | **Times variable selected** | **Number of linear fits** | **sHR [CI_sHR_]^1^ range^2^** | |
| --- | --- | --- | --- | --- | --- |
|  |  |  |  | **min** | **max** |
| Age [year] | 10 | 10 | 10 | 1.0443 [1.0286; 1.0601] | 1.0548 [1.0380; 1.0718] |
| CCI^3^ | 10 | 10 | 10 | 1.1045 [1.0173; 1.1992] | 1.1751 [1.0825; 1.2755] |
| Gender [ref male] | 10 | 10 | 10 | 1.4662 [1.0443; 2.0585] | 1.6563 [1.1806; 2.3237] |
| Vascular access [ref catheter] | 10 | 10 | 10 | 0.6078 [0.4271; 0.8648] | 0.6821 [0.4825; 0.9644] |
| Dialysis vintage [month] | 10 | 10 | 4 | 1.0035 [1.0018; 1.0053] | 1.0046 [1.0029; 1.0064] |
| Dialysis modality [ref HD] | 10 | 2 | 2 | 1.6872 [0.8754; 3.2517] | 1.7491 [0.8396; 3.6435] |

^1^CI_sHR_ = confidence interval of subdistribution hazard ratio; ^2^only indicated for linear models; ^3^CCI = Charlson comorbidity index

**Table S2. T50 and secondary outcomes**

| **Outcome** | **Number of runs** | **Times T50 selected** | **Number of linear fits** | **Mean c statistic** | **sHR [CI_sHR_]^1^ range^2^** | |
| --- | --- | --- | --- | --- | --- | --- |
|  |  |  |  |  | **min** | **max** |
| CV-related mortality | 5 | 0 | 0 | 0.5 | ND^3^ | ND |
| All-cause hospitalization | 10 | 7 | 7 | 0.5284 | 0.9976 [0.9960; 0.9991] | 0.9982 [0.9967; 0.9998] |
| CV-related hospitalization | 5 | 0 | 0 | 0.5 | ND | ND |

^1^CI_sHR_ = confidence interval of subdistribution hazard ratio; ^2^only indicated for linear models; ^3^ND = not determined

**Table S3. Calcification related parameters and outcomes**

| **Outcome** | **Total runs** | **Variable selection (times selected across total runs)** | | | | | | **Mean c statistic** |
| --- | --- | --- | --- | --- | --- | --- | --- | --- |
|  |  | **Albumin** | **P** | **Ca** | **Mg** | **Bic** | **Fetuin-A** |  |
| All-cause mortality | 5 | 5 | 0 | 1 | 0 | 0 | 1 | 0.6211 |
| All-cause hospitalization | 5 | 3 | 0 | 0 | 0 | 2 | 2 | 0.5406 |

P = phosphate; Ca = Calcium; Mg = Magnesium; Bic = Bicarbonate

**Table S4. Calcification related parameters & T50 and outcomes**

| **Outcome** | **Total runs** | **Variable selection (times selected across total runs)** | | | | | | | **Mean c statistic** |
| --- | --- | --- | --- | --- | --- | --- | --- | --- | --- |
|  |  | **Albumin** | **P** | **Ca** | **Mg** | **Bic** | **Fetuin-A** | **T50** |  |
| All-cause mortality | 5 | 5 | 0 | 1 | 0 | 0 | 1 | 0 | 0.6211 |
| All-cause hospitalization | 5 | 3 | 0 | 0 | 0 | 2 | 2 | 0 | 0.5406 |

P = phosphate; Ca = Calcium; Mg = Magnesium; Bic = Bicarbonate


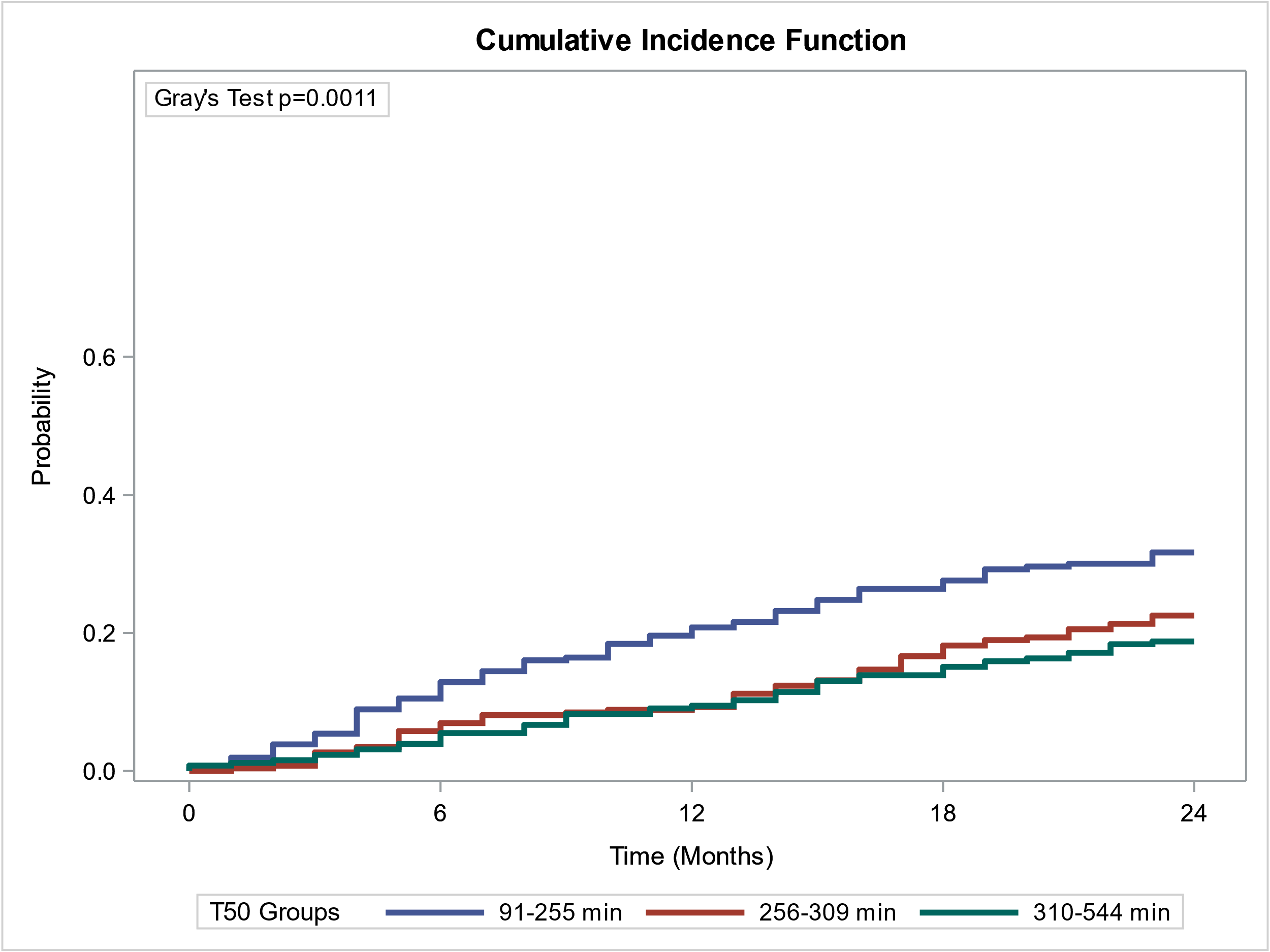


**Figure S1: Cumulative Incidence Function for T50 groups**
